# Supplementary material for: Ozone Pollution Alters Olfaction and Behavior of Pollinators
Source: Antioxidants (Basel). 2021 Apr 21;10(5):636. doi: 10.3390/antiox10050636 (PMC8143334; doi:10.3390/antiox10050636)
Supplement: Supplementary file 1 [file antioxidants-10-00636-s001.zip › antioxidants-1176102-supplementary.pdf]

antioxidants

MDPI

Supplementary Materials

Ozone pollution alters olfaction and behavior of pollinators

Maryse Vanderplanck [1,2,*], Benoît Lapeyre [3], Margot Brondani [3], Manon Opsommer [3], Mathilde Dufay [3], Martine Hossaert-McKey [3,4], Magali Proffit [3]

[1] Université de Lille, CNRS, UMR 8198 – Evo-Eco-Paléo, 59000 Lille, France
[2] Université de Mons, Laboratoire de Zoologie, 7000 Mons, Belgium
[3] Centre d'Écologie Fonctionnelle et Évolutive (CEFE), Université de Montpellier, CNRS, Université Paul Valéry Montpellier 3, EPHE, IRD, 34293 Montpellier, France
[4] Laboratoire de Chimie Bio-inspirée et Innovations Écologiques (CHIMECO), CNRS – Université de Montpellier, 34790 Grabels, France
* Correspondence: maryse.vanderplanck@umons.ac.be, +32 65 37 38 93

Table S1. Conditions of ozone exposure and sample sizes for electrophysiological experiments.

Pollinator species	Tested VOCs	Exposure duration (min)	O_3 concentration (ppb)	Sample size (number of individuals)
Blastophaga psenes	Linalool mixture, Linalool oxides, Benzyl alcohol	60	0	21
			200	17
		180	0	12
			80	11
			120	11
Bombus terrestris	R-linalool, Benzaldehyde, Nonanal	60	0	30
			80	30
			120	30
			200	30
		180	0	30
			80	30
			120	30
			200	30

Table S2. Provider, CAS (Chemical Abstracts Service) number and purity of the synthetic VOCs used during the experiments.

Synthetic VOCs	Models	Provider	CAS number	Purity
R-linalool	Bumblebee	Sigma-Aldrich	126-91-0	≥ 95 %
Linalool mixture	Fig wasp	Fluka	78-70-6	~ 97 %
Linalool oxides	Fig wasp	Fluka	68780-91-6	> 97 %
Benzyl alcohol	Fig wasp	Fluka	100-51-6	> 99.5 %
Benzaldehyde	Bumblebee	Sigma-Aldrich	100-52-7	≥ 99.5 %
Nonanal	Bumblebee	Sigma-Aldrich	124-19-6	≥ 99.5 %

Table S3. Conditions of ozone exposure and sample sizes for behavioral assays.

Pollinator species	Tested VOCs	Exposure duration (min)	O_3 concentration (ppb)	Sample size (number of individuals)
Blastophaga psenes	VOCs mix mimicking the odour of the fig host	60	0	49
			120	50
			200	50
		180	0	50
			80	50
			120	50
Bombus terrestris	Benzaldehyde	60	0	30
			80	30
			120	30
			200	30

Table S4. Statistical outputs (p-values) regarding the impact of ozone exposure on antennal sensitivity in fig wasps. Significant differences are in bold.

Comparison between 0 and 200 ppb for 60-min exposure				
VOCs	VOC dose (µg)			
	1	10	100	1000
Benzyl alcohol	0.850	0.928	0.067	**0.017**
Linalool oxides	0.956	0.782	0.190	0.133
Linalool mixture	0.753	0.856	0.117	0.187

Multiple pairwise comparisons for 180-min exposure					
VOCs	Ozone concentrations	VOC dose (µg)			
		1	10	100	1000
Benzyl alcohol	0 ppb vs 80 ppb	0.079	0.081	0.812	0.238
	0 ppb vs 120 ppb	**0.018**	0.064	0.198	0.431
	80 ppb vs 120 ppb	0.535	0.917	0.304	0.700
Linalool oxides	0 ppb vs 80 ppb	0.289	0.560	0.960	0.229
	0 ppb vs 120 ppb	0.945	0.064	0.511	0.450
	80 ppb vs 120 ppb	0.331	0.211	0.489	0.056
Linalool mixture	0 ppb vs 80 ppb	0.641	**0.026**	0.478	0.625
	0 ppb vs 120 ppb	0.187	**0.007**	0.809	0.885
	80 ppb vs 120 ppb	0.081	0.623	0.647	0.737

Table S5. Statistical outputs (p-values) regarding the impact of ozone exposure on antennal sensitivity in bumblebee workers. Significant differences are in bold.

Multiple pairwise comparisons for 60-min exposure					
		VOC dose (µg)			
	Ozone concentrations	1	10	100	1000
Benzaldehyde	0 ppb vs 80 ppb	0.883	0.686	0.769	0.907
	0 ppb vs 120 ppb	0.574	**0.023**	**0.002**	**0.026**
	0 ppb vs 200 ppb	0.658	0.244	**0.048**	**0.045**
	80 ppb vs 120 ppb	0.678	0.061	**0.006**	**0.034**
	80 ppb vs 200 ppb	0.768	0.446	0.092	0.059
	120 ppb vs 200 ppb	0.905	0.265	0.275	0.819
R-Linalool	0 ppb vs 80 ppb	0.723	0.460	0.455	0.663
	0 ppb vs 120 ppb	0.527	0.111	**0.012**	**0.014**
	0 ppb vs 200 ppb	0.426	0.122	**0.014**	**0.004**
	80 ppb vs 120 ppb	0.781	0.392	0.077	**0.044**
	80 ppb vs 200 ppb	0.658	0.419	0.086	**0.013**
	120 ppb vs 200 ppb	0.869	0.961	0.956	0.643
Nonanal	0 ppb vs 80 ppb	0.752	0.956	0.816	0.380
	0 ppb vs 120 ppb	0.874	0.687	**0.043**	0.300
	0 ppb vs 200 ppb	0.839	0.526	**0.037**	0.159
	80 ppb vs 120 ppb	0.875	0.728	0.074	0.056
	80 ppb vs 200 ppb	0.604	0.563	0.064	**0.022**
	120 ppb vs 200 ppb	0.718	0.817	0.950	0.708

Multiple pairwise comparisons for 180-min exposure					
		VOC dose (µg)			
	Ozone concentrations	1	10	100	1000
Benzaldehyde	0 ppb vs 80 ppb	0.915	0.814	0.243	0.144
	0 ppb vs 120 ppb	0.943	0.524	0.077	0.052
	0 ppb vs 200 ppb	0.980	0.386	**0.031**	**0.012**
	80 ppb vs 120 ppb	0.859	0.688	0.549	0.629
	80 ppb vs 200 ppb	0.935	0.527	0.319	0.292
	120 ppb vs 200 ppb	0.923	0.818	0.691	0.568
R-Linalool	0 ppb vs 80 ppb	0.947	0.993	0.291	**0.025**
	0 ppb vs 120 ppb	0.904	0.622	0.074	0.082
	0 ppb vs 200 ppb	0.764	0.421	**0.032**	**0.003**
	80 ppb vs 120 ppb	0.851	0.616	0.462	0.611
	80 ppb vs 200 ppb	0.713	0.416	0.274	0.490
	120 ppb vs 200 ppb	0.857	0.755	0.720	0.231

Nonanal	0 ppb vs 80 ppb	0.796	0.757	0.695	0.538
	0 ppb vs 120 ppb	0.955	0.919	0.436	0.737
	0 ppb vs 200 ppb	0.801	0.868	0.294	0.491
	80 ppb vs 120 ppb	0.753	0.681	0.699	0.780
	80 ppb vs 200 ppb	0.996	0.887	0.511	0.941
	120 ppb vs 200 ppb	0.757	0.788	0.786	0.724
